# Strategies Older Adults Used During COVID-19 to Stay Connected

**DOI:** 10.1093/geroni/igab046.082

**Published:** 2021-12-17

**Authors:** Brad Doebbeling, Haley Harrelson, Michelle Houchins, Hallie Wine, Claire Pishko, Cheryl Der Ananian, Allie Peckham, M Aaron Guest

**Affiliations:** 1 College of Health Solutions, Arizona State University, Phoenix, Arizona, United States; 2 Arizona State University, Phoenix, Arizona, United States; 3 Arizona State University, Pheonix, Arizona, United States

## Abstract

An unintended consequence of the physical distancing guidelines to prevent the spread of COVID-19 may be increased feelings of social isolation and loneliness in older adults. Purpose: The purpose of this study was to identify successful strategies used in avoiding social isolation and feelings of loneliness in older adults (50+) during the pandemic. Methods: Older adults (n=22) selected from a longitudinal study, Aging In the Time of COVID-19, who did not report loneliness, participated in a semi-structured interview via zoom. Individuals were asked a series of questions about how their lives were impacted by the pandemic and what they did to avoid social isolation and loneliness. Each interview was recorded and transcribed verbatim. transcripts were analyzed and categorized to identify common strategies. Results: Participants were primarily female and white (100%) with a mean age of 64.7 years. Preliminary findings (n=5) suggest older adults developed several effective strategies for combating social isolation and feelings of loneliness, including purposely reaching out and “checking in” on others; engaging in exercise, either alone or with others, and engaging in outdoor activities, such as socially distanced in-person encounters. Other effective strategies included virtual events (e.g. community or local events, museums or concerts, etc.), using technology to communicate with friends and family, and practicing gratitude consistently. Conclusions: Although older adults have been encouraged to stay at home and physically distanced throughout the pandemic, they have found ways to remain socially connected with friends, family, and community, despite not being physically together.

